# MicroRNA-145 induces apoptosis of glioma cells by targeting BNIP3 and Notch signaling

**DOI:** 10.18632/oncotarget.18604

**Published:** 2017-06-22

**Authors:** Yan Du, Juan Li, Tao Xu, Dan-Dan Zhou, Lei Zhang, Xiao Wang

**Affiliations:** ^1^ School of Pharmacy, Anhui Province Key Laboratory of Major Autoimmune Diseases, Anhui Institute of Innovative Drugs, Anhui Medical University, Hefei 230032, China; ^2^ Institute for Liver Disease of Anhui Medical University, Anhui Medical University, Hefei 230032, China; ^3^ Anhui Provincial Hospital, Hefei 230032, China; ^4^ Department of Radiology, The First Affiliated Hospital of Anhui Medical University, Hefei 230022, China

**Keywords:** malignant gliomas, BNIP3, miR-145, apoptosis, notch signaling

## Abstract

MicroRNAs (miRNAs) are involved in the pathogenesis of various human cancers. Here we show that miR-145 expression is decreased in human glioma samples, rat glioma tissues, and glioma cell lines, while expression of BNIP3 is increased. Over-expression of miR-145 or suppression of BNIP3 induced glioma cell apoptosis. BNIP3 is localized in the nucleus in glioma cells, and miR-145 inhibits BNIP3 expression by binding to the 3’ untranslated region of its mRNA. Interestingly, miR-145 and BNIP3 regulate glioma cell apoptosis by modulating Notch signaling. These results indicate that miR-145 increases glioma cell apoptosis by inhibiting BNIP3 and Notch signaling, and suggest that miR-145 may serve as a novel therapeutic target for malignant glioma.

## INTRODUCTION

Malignant glioma is one of the most aggressive and common primary central nervous system tumors with high mortality and poor 5-year survival rate [1]. Based on the degree of malignancy, gliomas are divided into four histopathologic grades [2]. Glioblastoma multiforme (GBM) is characterized by diffuse invasion, apoptosis resistance, robust angiogenesis, and an immature profile with developmental plasticity [3]. GBM usually spreads quickly and invades other parts of the brain with tentacle-like projections, making a complete surgical removal difficult.

MicroRNAs (miRNAs) are a class of small 20–22 nucleotide-long non-coding RNAs that regulate growth, invasion, and cell cycle of cancer cells [4-7]. In multiple cellular processes, including development, proliferation, and differentiation, miRNAs fine-tune the cellular fate by targeting important transcription factors and key pathways. Impairment of the miRNA regulatory network has been proposed as one of the key mechanisms in GBM pathogenesis [8]. MiR-145 is a putative tumor suppressor miRNA that is down-regulated in various types of cancers [4, 9]. However, its role in malignant gliomas remains to be elucidated.

Notch signaling plays a pivotal role in human cancers, including malignant gliomas, by promoting glioma cells self-renewal and suppressing their differentiation [10-13]. The Notch intracellular domain translocates into the nucleus and induces transcription of its target genes, including genes in the hairy/enhancer of split (Hes) and Hes-related with YRPW motif (Hey) families [14]. It was reported that the tumor suppressors miR-143 and miR-145 could modulate vascular smooth muscle cell differentiation by inactivating Notch-1 signaling [15], but the specific role of miR-145 in regulating the Notch signaling in malignant glioma is unknown.

Bcl2/adenovirus E1b 19-kDa interacting protein 3 (BNIP3) is a BH3-only protein that is localized in the mitochondria and contributes to ischemia-reperfusion (I/R) injury by inducing mitochondrial dysfunction [16-19]. BNIP3 is up-regulated by the transcription factor HIF-1 in hypoxic regions of tumors [20, 21]. In tumors, BNIP3 is localized mainly in the nucleus, and inhibits apoptosis [22]. A recent study has indicated that the nuclear BNIP3 forms a complex with histone deacetylase 1 (HDAC1) and PTB-associating splicing factor (PSF) to down-regulate AIF expression in glioma cells, leading to their resistance to temozolomide-induced cell death [23]. In addition, BNIP3 can block TRAIL- and hypoxia-induced apoptosis in GBM tumors [24, 25]. However, the Notch regulation by BNIP3 in glioma cells has not been investigated.

In this study, we have investigated the role of miR-145 in glioma cells. Our results demonstrate that BNIP3 is localized in the nucleus of glioma cells, and serves as a target of miR-145. MiR-145 promotes apoptosis of glioma cells by inhibiting BNIP3, resulting in the inhibition of Notch signaling.

## RESULTS

### Glioma MRI evaluation in rats

**Figure 1:**
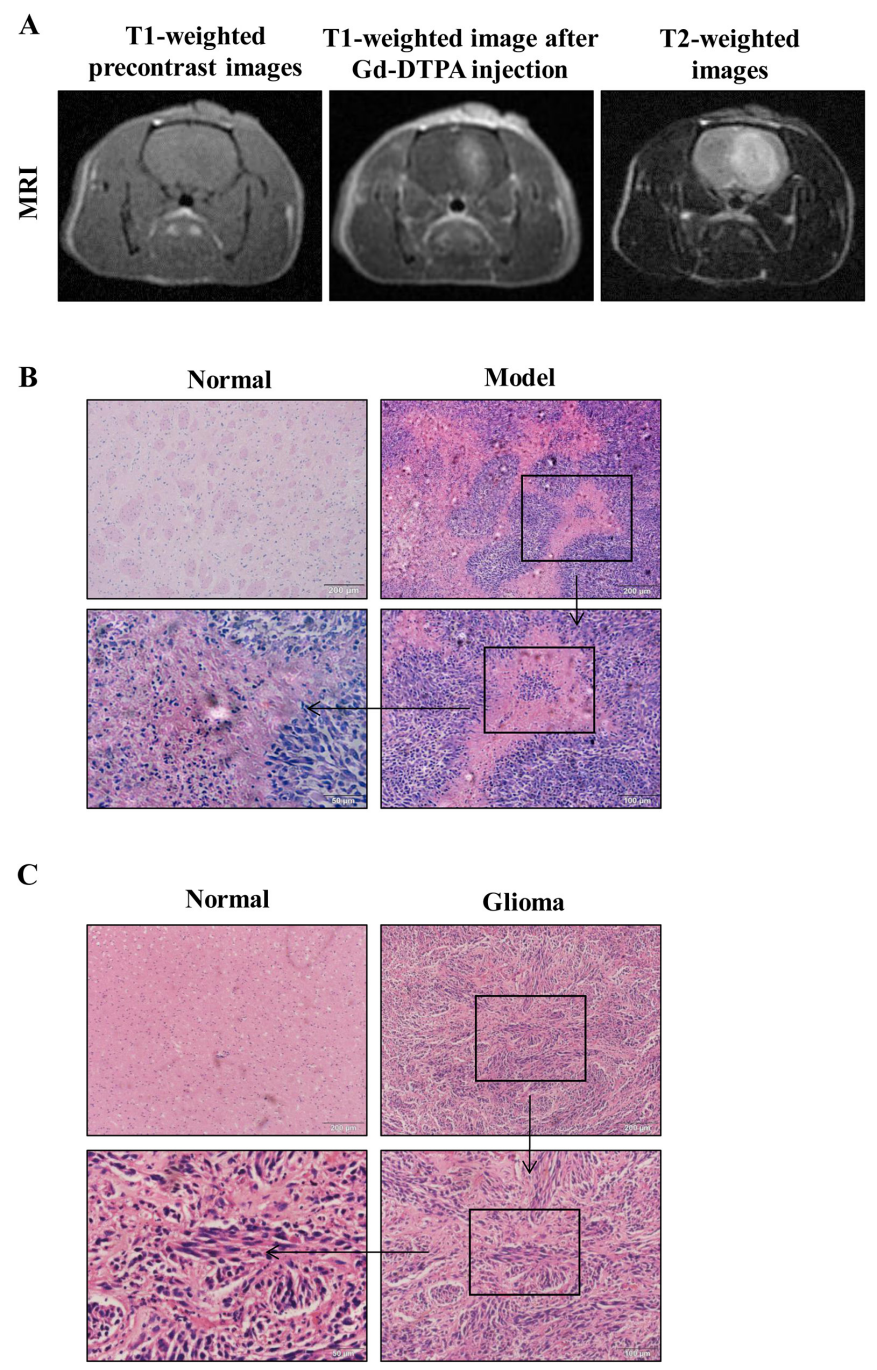
MRI and corresponding histopathological findings **(A)** MR images of a tumor in the rat brain glioma tissue. **(B)** Pathology observation of rat brain tissues sections stained with hematoxylin and eosin (H&E) (×100, ×200, × 400).

First, we evaluated glioma formation in rats by implanting rats with glioma C6 cells, and evaluating the tumor development by MRI. MRI is a particularly attractive technique for glioma evaluation because it is fast and noninvasive. The tumor foci started to develop 5 days after implantation, and appeared as enhanced distinct tumor foci on day 7. From a macroscopic point of view, T1-weighted pre-contrast images showed mild hypo-intensity signal tumors with ill-defined boundaries. After Gd-DTPA injection, the tumors showed intense enhancement with blurring margins on day 7 (Figure 1A). They grew rapidly and enlarged to occupy the most portion of the left cerebral hemisphere at the end of the 3rd week after implantation. T2-weighted images showed hyper-intensity signal feature of the tumors (Figure 1A). Implantation with C6 cells produced brain tumors in 6 out of 10 rats. The masses exhibited the properties of malignant tumors, including the presence of pseudo-palisading cells and microvascular proliferation, compared to normal tissues (Figure 1B). The pathological information of the patient samples was proved grade gliomas (WHO grade III) by Histopathology (Figure 1C).

### MiR-145 expression is decreased in gliomas

**Figure 2:**
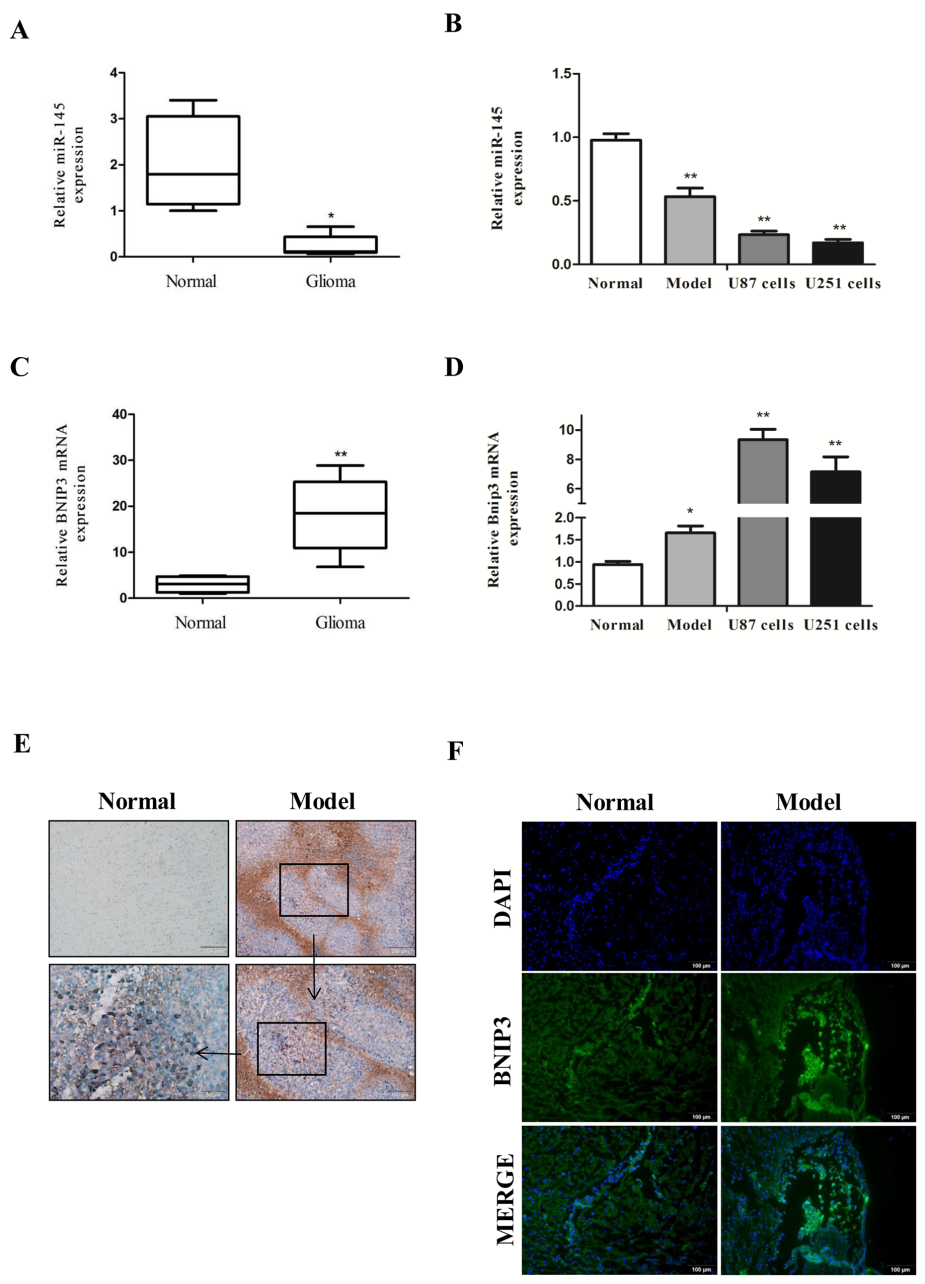
Down-regulation of miR-145 and up-regulation of BNIP3 expression in gliomas **(A)** Quantitative real-time PCR of miR-145 expression in glioma samples (n = 19) compared with normal samples (n = 10). **(B)** quantitative real-time PCR of miR-145 expression in rat glioma tissues (n = 6) and U87, U251 glioma cells compared to normal tissues (n = 6). **(C)** Quantitative real-time PCR of BNIP3 mRNA expression in glioma samples (n = 19) compared with normal samples (n = 10). **(D)** quantitative real-time PCR of BNIP3 mRNA expression in rat glioma tissues and U87, U251 glioma cells compared to normal tissues. **(E)** Pathology observation of mice brain tissues sections stained with IHC (×100, ×200, ×400). **(F)** Immunofluorescence with BNIP3 (green) in rat normal tissues and glioma tissues (×200).*p < 0.05, **p < 0.01 versus control group.

To define the role of miR-145 in glioma, we analyzed miR-145 levels in human glioma samples, rat glioma tissues, and glioma cell lines by quantitative real-time PCR analysis. We found that miR-145 levels were decreased in human glioma samples (WHO I/II, n=11 and WHO III/IV, n=8) compared with normal samples (n = 10) (Figure 2A). In addition, miR-145 levels were decreased in rat glioma tissues (n = 6), and glioma cell lines (U87 and U251) compared with rat normal brain tissues (n = 6) (Figure 2B).

### BNIP3 expression inversely correlates with miR-145 in gliomas

To define the role of BNIP3 in gliomas, we measured BNIP3 mRNA expression in glioma human samples, rat tissues, and glioma cell lines by quantitative real-time PCR. Compared with controls, BNIP3 mRNA levels in human glioma samples (Figure 2C), rat glioma tissues, and glioma cell lines (Figure 2D) were increased, suggesting that there may be a negative correlation between miR-145 and BNIP3.

To evaluate the protein levels of BNIP3, we analyzed the rat glioma tissues (n=6) by immunohistochemistry (IHC). Compared to control brain tissues, the expression of BNIP3 in rat glioma tissues was increased (Figure 2E; magnification: 100, 200, 400).

**Figure 3:**
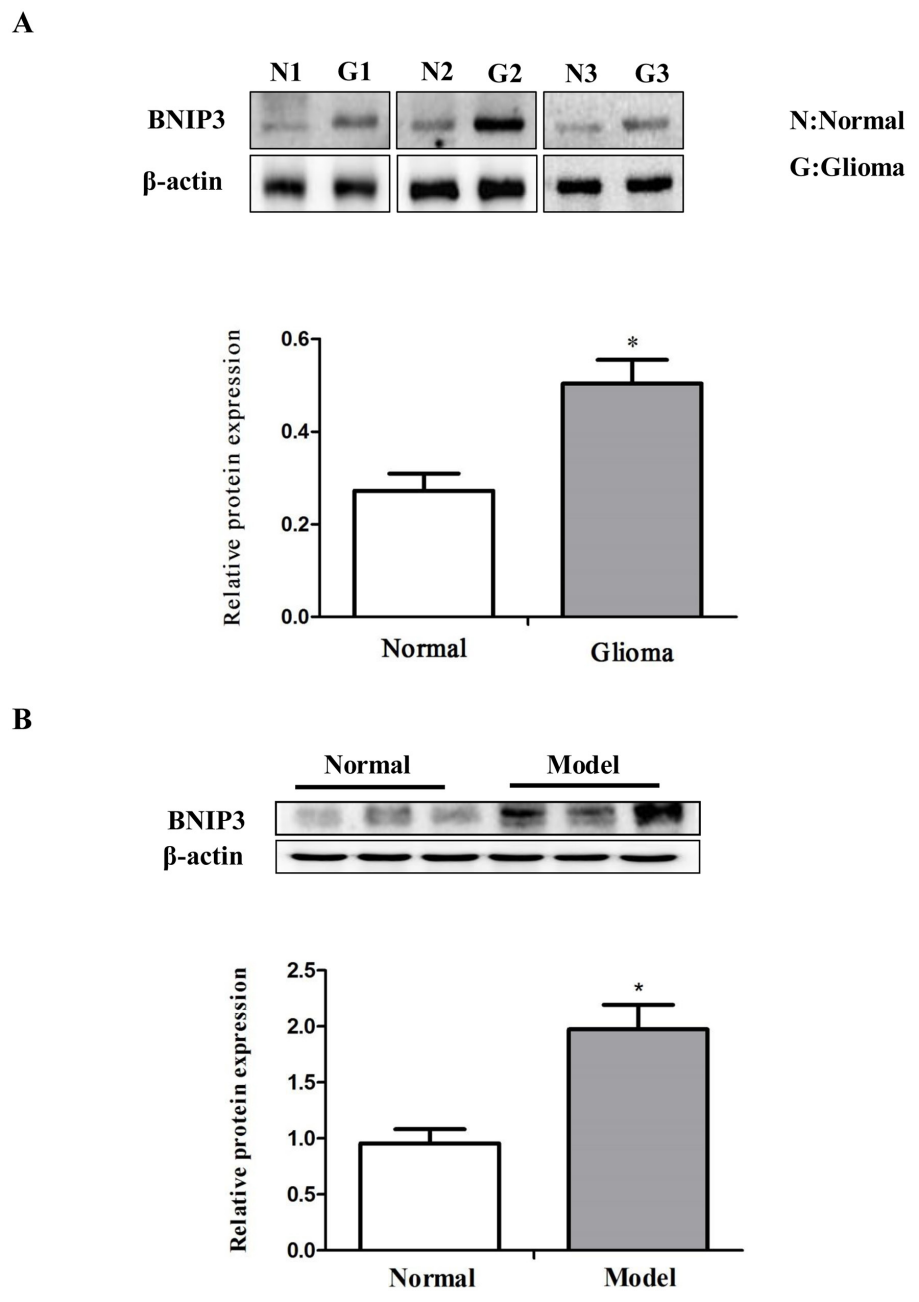
Down-regulation of miR-145 and up-regulation of BNIP3 expression in gliomas **(A)** Western analysis of BNIP3 and control β-actin in glioma samples compared with normal samples. **(B)** Western analysis of BNIP3 and control β-actin in rat glioma tissues compared to normal tissues. *p < 0.05, **p < 0.01 versus control group.

BNIP3 is a hypoxia-inducible pro-apoptotic member of the Bcl-2 family that induces cell death by associating with the mitochondria. However, in glioma cells, BNIP3 is localized in the nucleus, and inhibits apoptosis [22]. Thus, we analyzed whether BNIP3 is localized in the nucleus in rat glioma tissues to form gliomas. Immunofluorescence revealed abundant BNIP3 staining in the nucleus of glioma tissues (Figure 2F). Western analysis demonstrated that BNIP3 expression was higher in human glioma samples compared with normal samples (Figure 3A), and in rat glioma tissues and glioma cell lines compared with rat normal brain tissues (Figure 3B).

### miR-145 induces apoptosis of glioma cells

**Figure 4:**
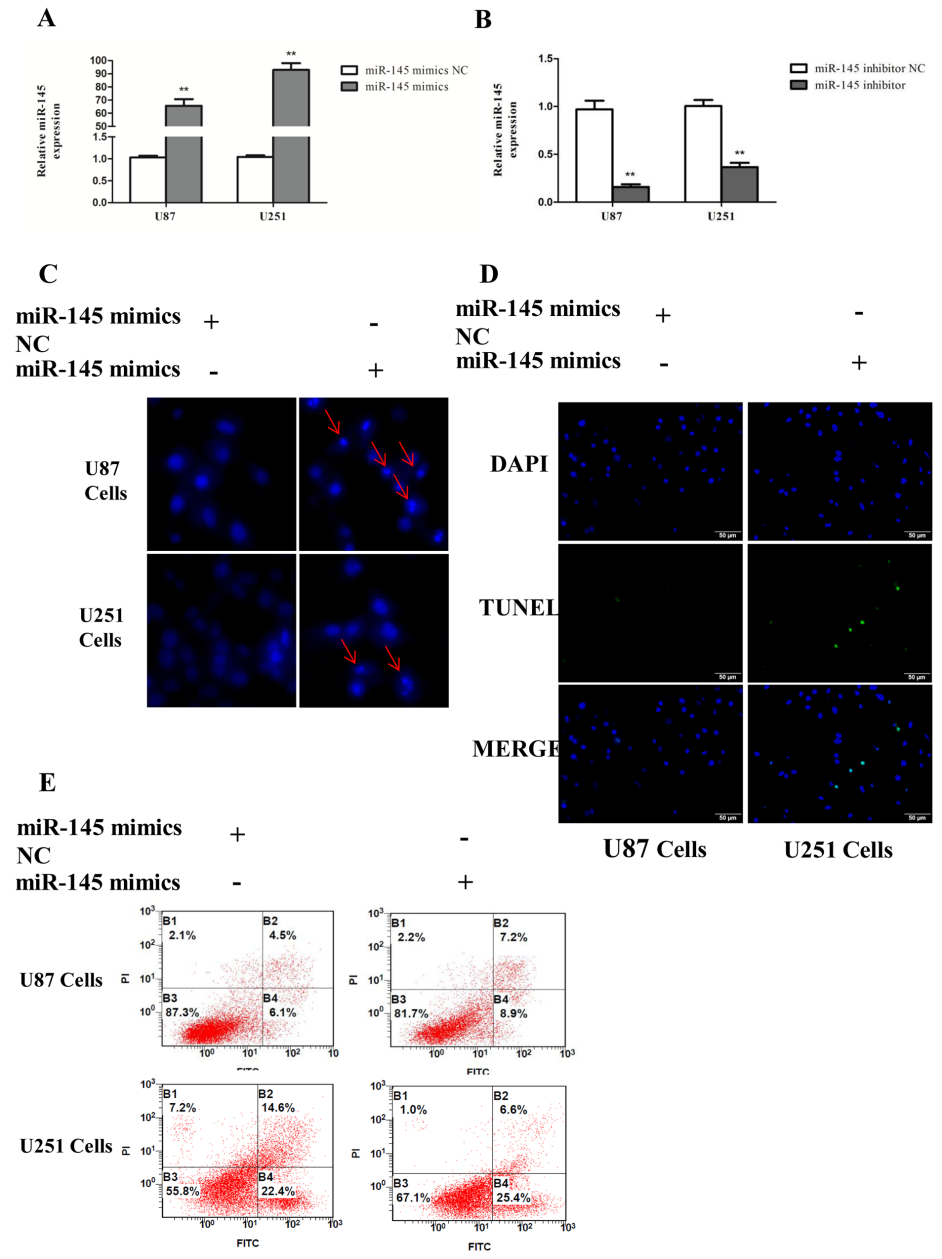
miR-145 induces glioma cells apoptosis **(A, B)** Transfection effect of miR-145 mimics or inhibitor was confirmed by quantitative real-time PCR. **(C)** U87 and U251 cells were stained with Hoechst 33342 dye after miR-145 mimics or mimics NC treatment. **(D)** U87 and U251 cells were stained with Tunel after miR-145 mimics or mimics NC treatment. **(E)** U87 cells apoptosis level after miR-145 mimics or mimics NC treatment was determined by FACS. *p < 0.05, **p < 0.01 versus control group.

To investigate the potential function of miR-145 in glioma cells apoptosis, U87 and U251 cells were transiently transfected with miR-145 mimics or inhibitor. Quantitative real-time PCR confirmed that miR-145 expression was increased after miR-145 mimics transfection, and decreased after miR-145 inhibitor transfection (Figure 4A, 4B). Apoptosis was evaluated by using Hoechst 33342 staining (Figure 4C), Tunel (Figure 4D) and Annexin V-FITC/PI double staining (Figure 4E). Cells transfected with miR-145 mimics exhibited increased signs of apoptosis compared to cells transfected with control mimics.

**Figure 5:**
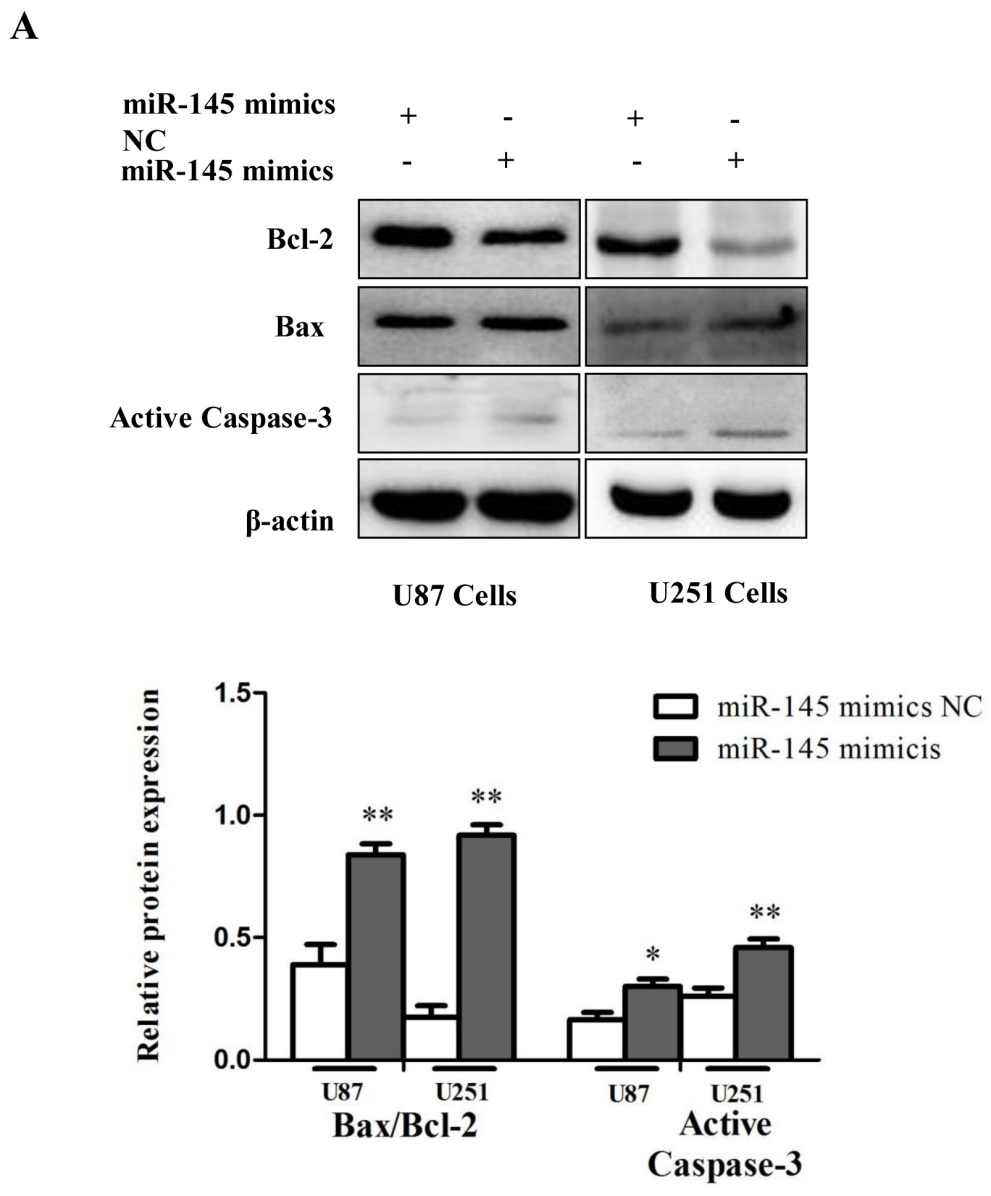
miR-145 induces glioma cells apoptosis **(A)** Western blot was performed to detect the expression levels of Bax, Bcl-2 and Active Caspase-3.

In addition, consistent with previous studies [26], cells transfected with miR-145 mimics exhibited decreased expression of the anti-apoptotic protein Bcl-2, increased expression of the pro-apoptotic proteins Bax, and Active Caspase-3, and an elevated Bax/Bcl-2 ratio (Figure 5A). Together, these results indicate that miR-145 induces apoptosis of glioma cells.

### miR-145 inhibits expression of BNIP3 via binding to its 3′-UTR

**Figure 6:**
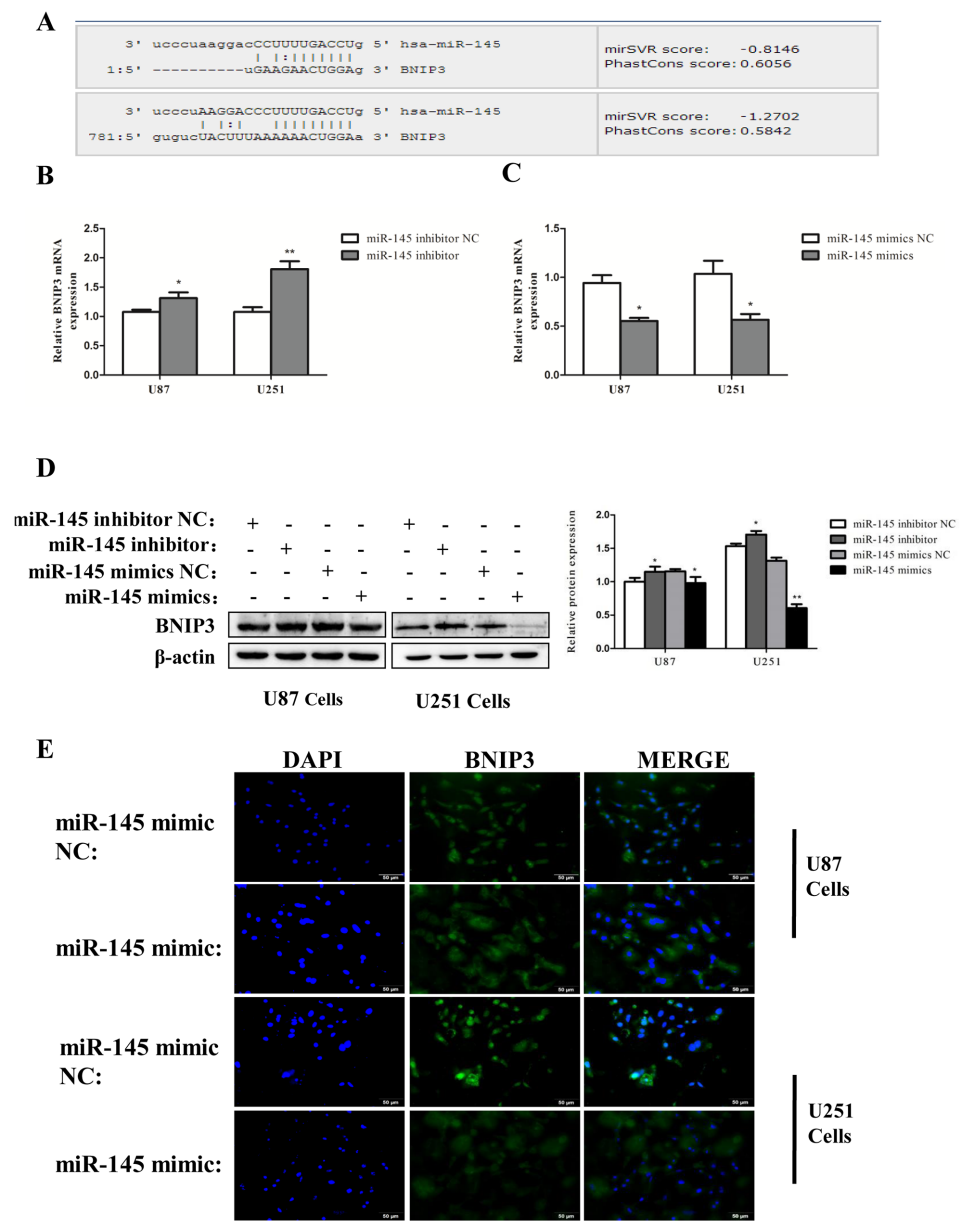
miR-145 inhibits mRNA and protein expression of BNIP3 **(A)** Bioinformatics analysis shows the seed sequence of miR-145 binding to the 3′-UTR of BNIP3 mRNA. **(B)**, **(C)** Quantitative real-time PCR analysis of mRNA expression of BNIP3 in U87 cells treated with miR-145 mimics and inhibitor for 48 h. **(D)** Western analysis of protein expression of BNIP3 in U87 and U251 cells treated with miR-145 mimics and inhibitor for 48 h. **(E)** Immunofluorescence with BNIP3 (green) in U87 and U251 cells after miR-145 mimics or mimics NC treatment.*p < 0.05, **p < 0.01 versus control group.

**Figure 7:**
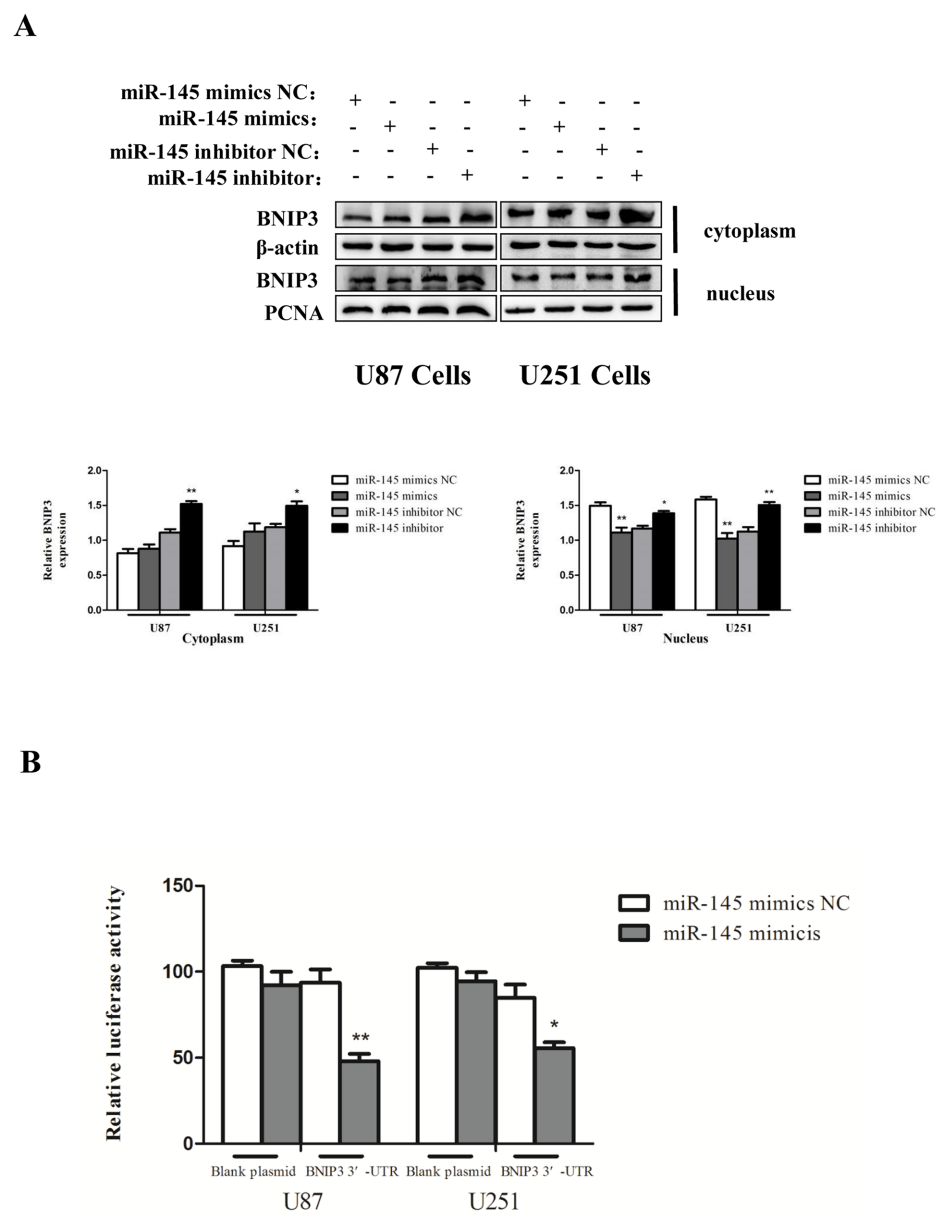
miR-145 inhibits mRNA and protein expression of BNIP3 **(A)** Western analysis of BNIP3, which is localized in the nucleus or the cytoplasm in U87 and U251 cells treated with miR-145 mimics and inhibitor for 48 h. **(B)** Wild-type 3′-UTR of BNIP3 gene was cloned into the firefly and Renilla reporter plasmid. The BNIP3-3′UTR constructs or blank plasmid were transfected into U87 and U251 cells with control or miR-145 mimics, followed by dual luciferase assays. *p < 0.05, **p < 0.01 versus control group.

We then searched for the target genes of miR-145 using microRNA.org and TargetScan. We found that the 3′-UTR of BNIP3 contains putative binding sites for miR-145 (Figure 6A). BNIP3 mRNA expression was decreased by miR-145 mimics (Figure 6B), and increased in cells transfected with miR-145 inhibitor (Figure 6C). Protein levels of BNIP3 also inversely correlated with miR-145 levels (Figure 6D). Immunofluorescence revealed that BNIP3 was localized in the nucleus of U87 and U251 cells, and its expression was decreased after miR-145 transfection (Figure 6E). Western blotting confirmed that the nuclear levels of BNIP3 were decreased in cells transfected with miR-145 mimics and increased in cells transfected with miR-145 inhibitor. Interestingly, the cytoplasmic levels of BNIP3 in cells transfected with miR-145 inhibitor were also increased (Figure 7A). To test if BNIP3 is a direct target of miR-145, the 3′-UTR was cloned into a luciferase expression vector to evaluate its response to miR-145. Co-transfection of luciferase reporter with the miR-145 mimics into U87 and U251 cells decreased expression of BNIP3 (Figure 7B), indicating that BNIP3 is a direct target of miR-145 in glioma cells.

### BNIP3 suppression induces apoptosis of glioma cells

**Figure 8:**
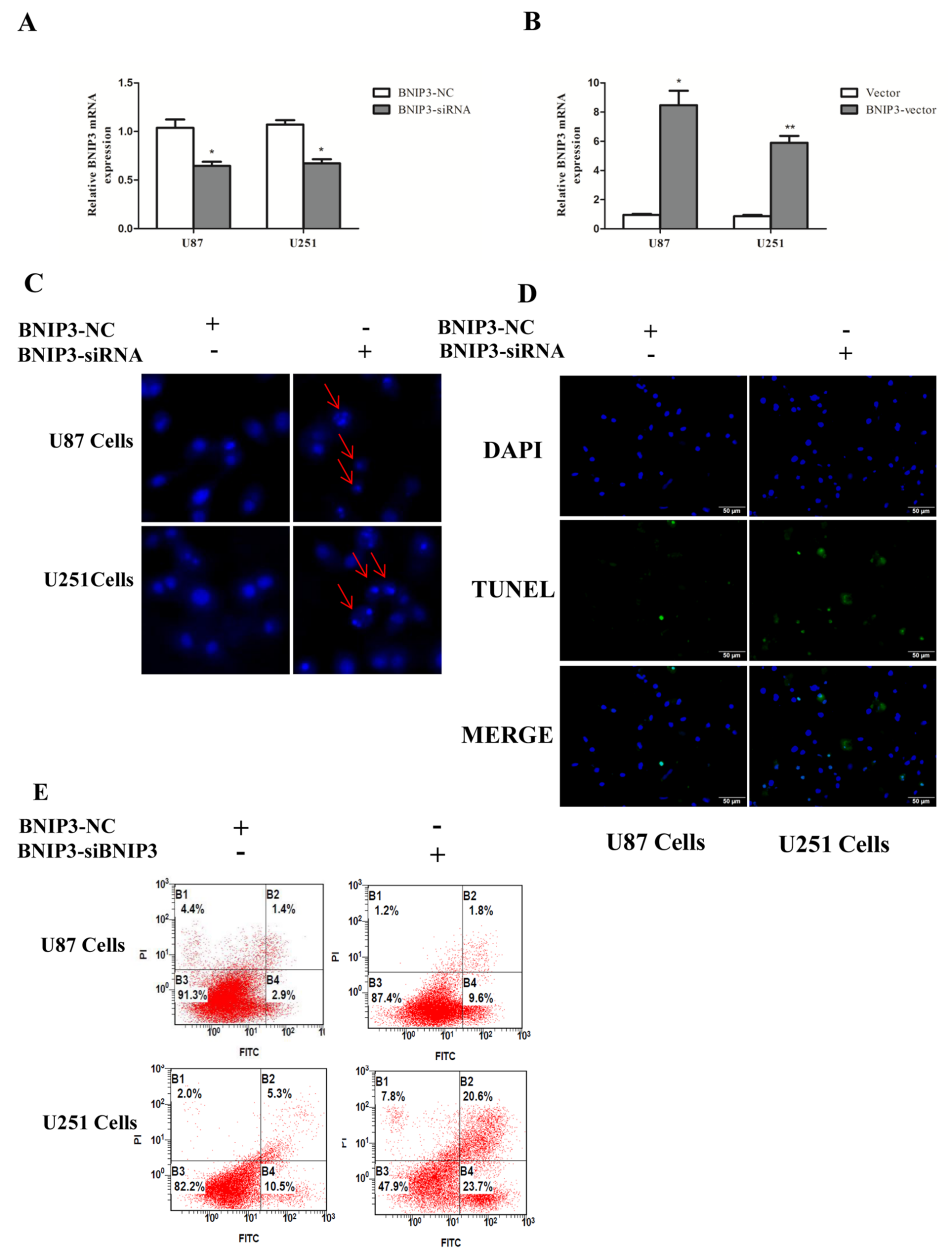
Knockdown of BNIP3 induces glioma cells apoptosis **(A, B)** Transfection effect of BNIP3-siRNA or BNIP3-vector was confirmed by quantitative real-time PCR. **(C)** U87 cells and U251 cells were stained with Hoechst 33342 dye after BNIP3-siRNA or control treatment. **(D)** U87 and U251 cells were stained with Tunel after BNIP3-siRNA or control treatment. **(E)** U87 cell apoptosis after BNIP3-siRNA or control treatment was determined by FACS. *p < 0.05, **p < 0.01 versus control group.

**Figure 9:**
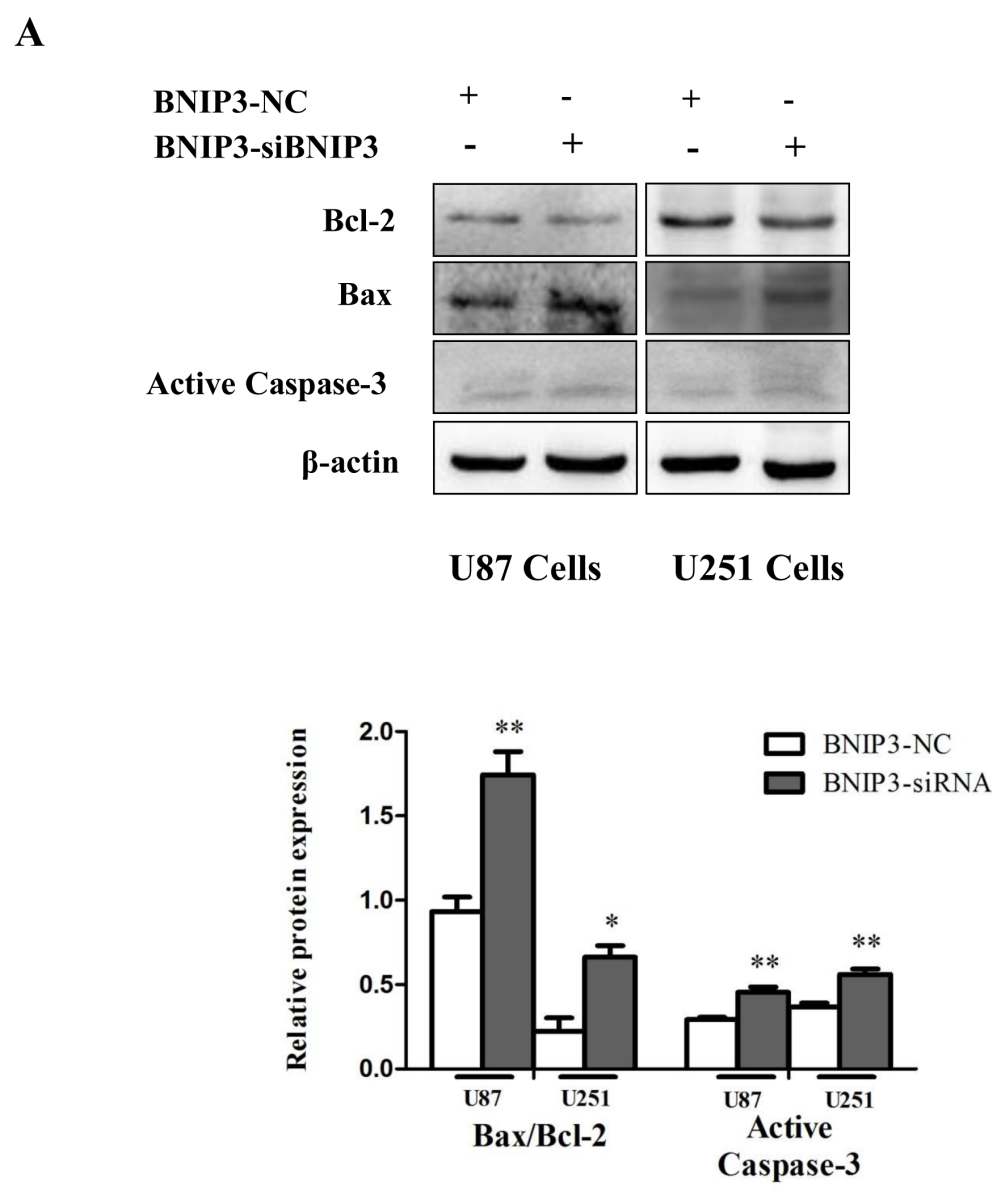
Knockdown of BNIP3 induces glioma cells apoptosis **(A)** Western blot was performed to detect the expression levels of Bax, Bcl-2 and Active Caspase-3.

In order to examine whether BNIP3 regulates glioma cells apoptosis, cells were transfected with BNIP3 siRNA or BNIP3-pEX-2 expression vector. Quantitative real-time PCR confirmed that BNIP3 expression was increased after BNIP3 expression vector transfection, and decreased after siRNA BNIP3 transfection (Figure 8A, 8B). Apoptosis assays using the Hoechst 33342 staining (Figure 8C), Tunel assay (Figure 8D), and Annexin V-FITC/PI double staining (Figure 8E) demonstrated that down-regulation of BNIP3 induced U87 and U251 cells apoptosis *in vitro*. In addition, cells transfected with BNIP3 siRNA exhibited decreased expression of Bcl-2, increased expression of Bax and Active Caspase-3, and increased Bax/Bcl-2 ratio (Figure 9A). Collectively, these results indicate that BNIP3 inhibits apoptosis of glioma cells.

### miR-145 inhibits Notch pathway by targeting BNIP3

**Figure 10:**
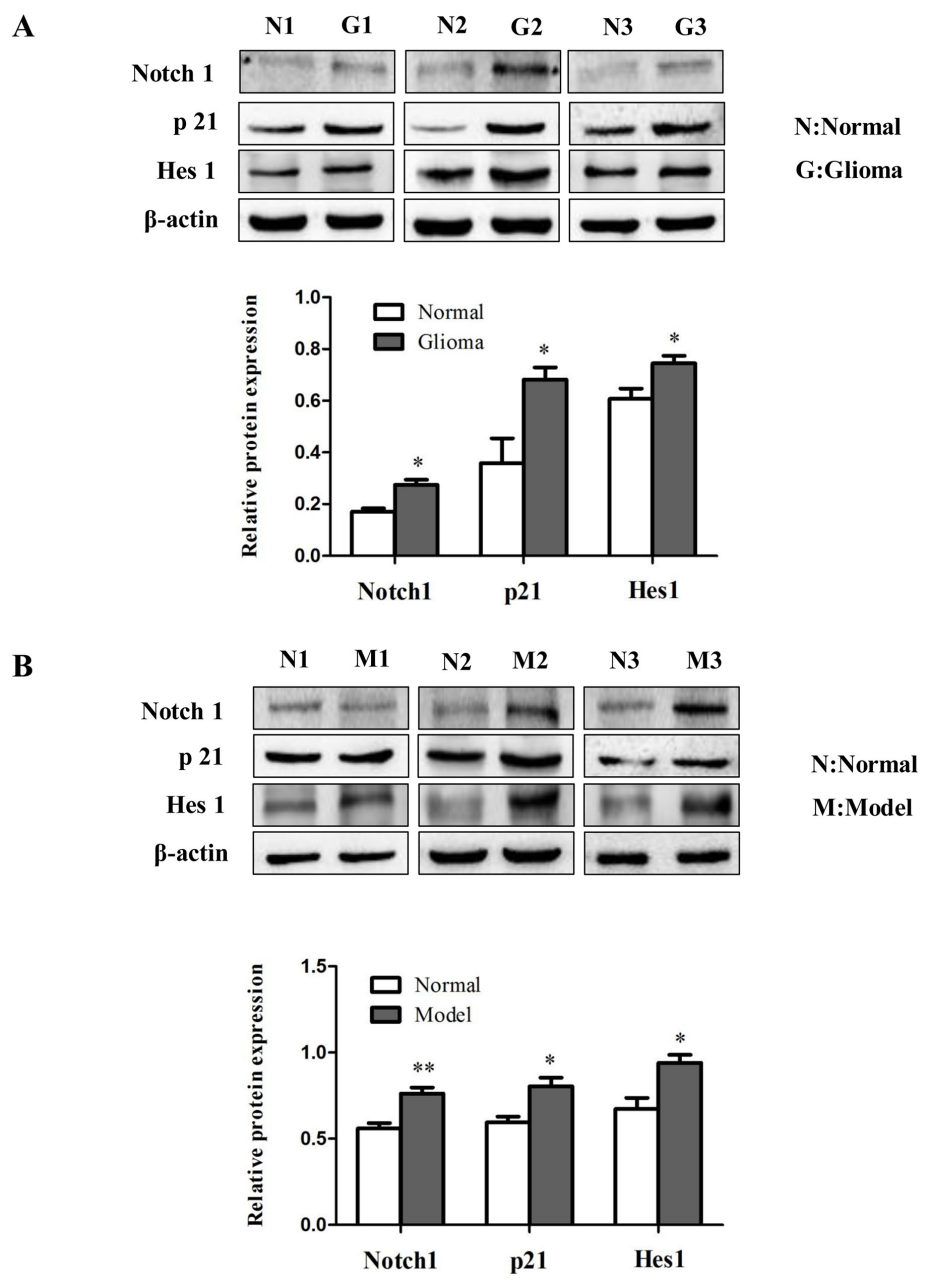
The role of Notch signaling in gliomas **(A)** The protein expression of Notch1, p21 and Hes1 was determined by western blot analysis in glioma samples compared with normal samples. **(B)** Western analysis of Notch signaling pathway related proteins in rat glioma tissues compared with normal tissues. *p < 0.05, **p < 0.01 versus control group.

**Figure 11:**
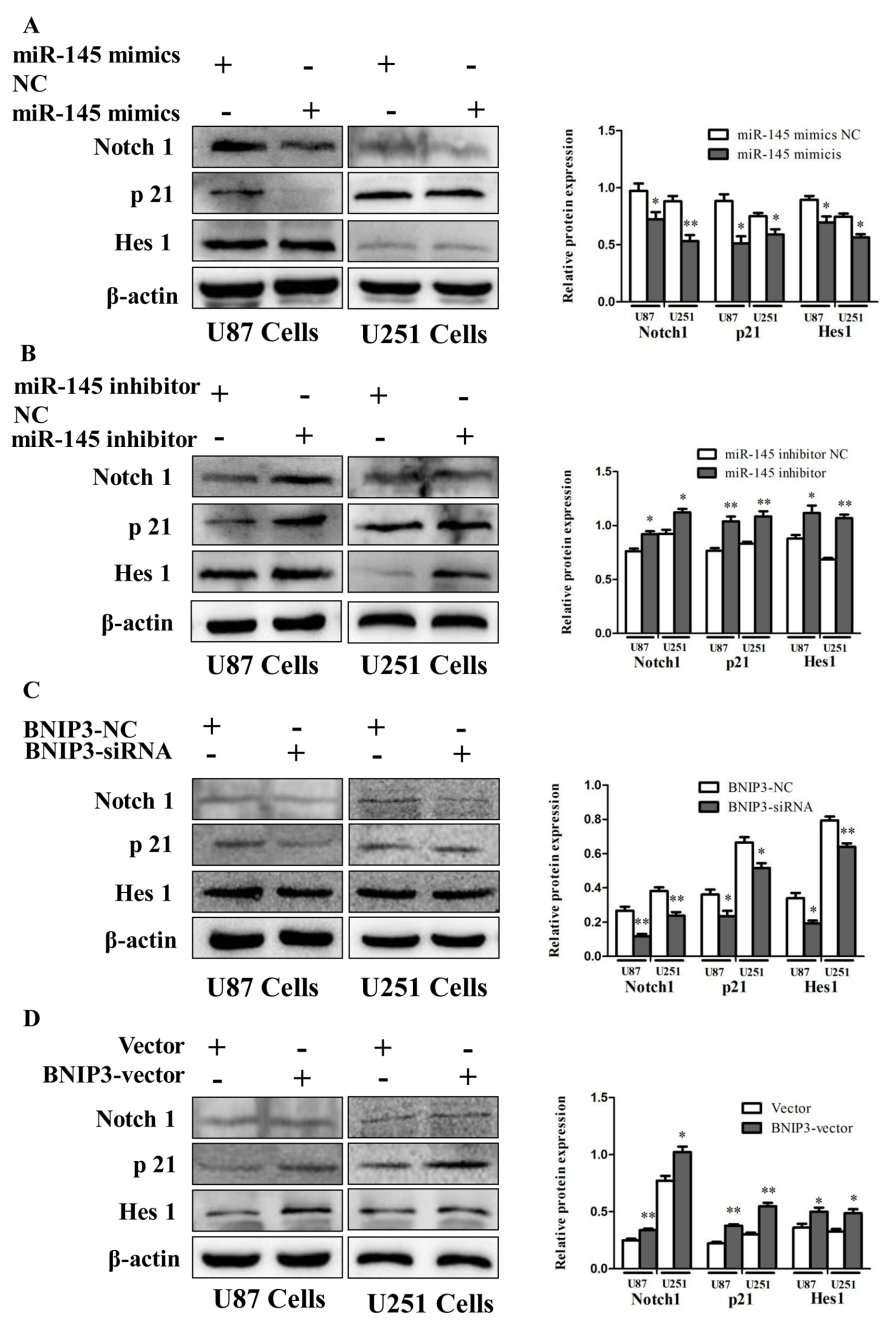
miR-145 regulates Notch signaling by targeting BNIP3 **(A, B)** Protein expression of Notch1, p21 and Hes1 was determined by western blot analysis in U87 and U251 cells transfected with miR-145 mimics and mimics-NC, or miR-145 inhibitor and inhibitor-NC. **(C, D)** Western analysis of Notch1-related proteins in U87 and U251 cells transfected with Bnip3 siRNA and siRNA control, or BNIP3 expression vector and blank vector. *p < 0.05, **p < 0.01 versus control group.

**Figure 12:**
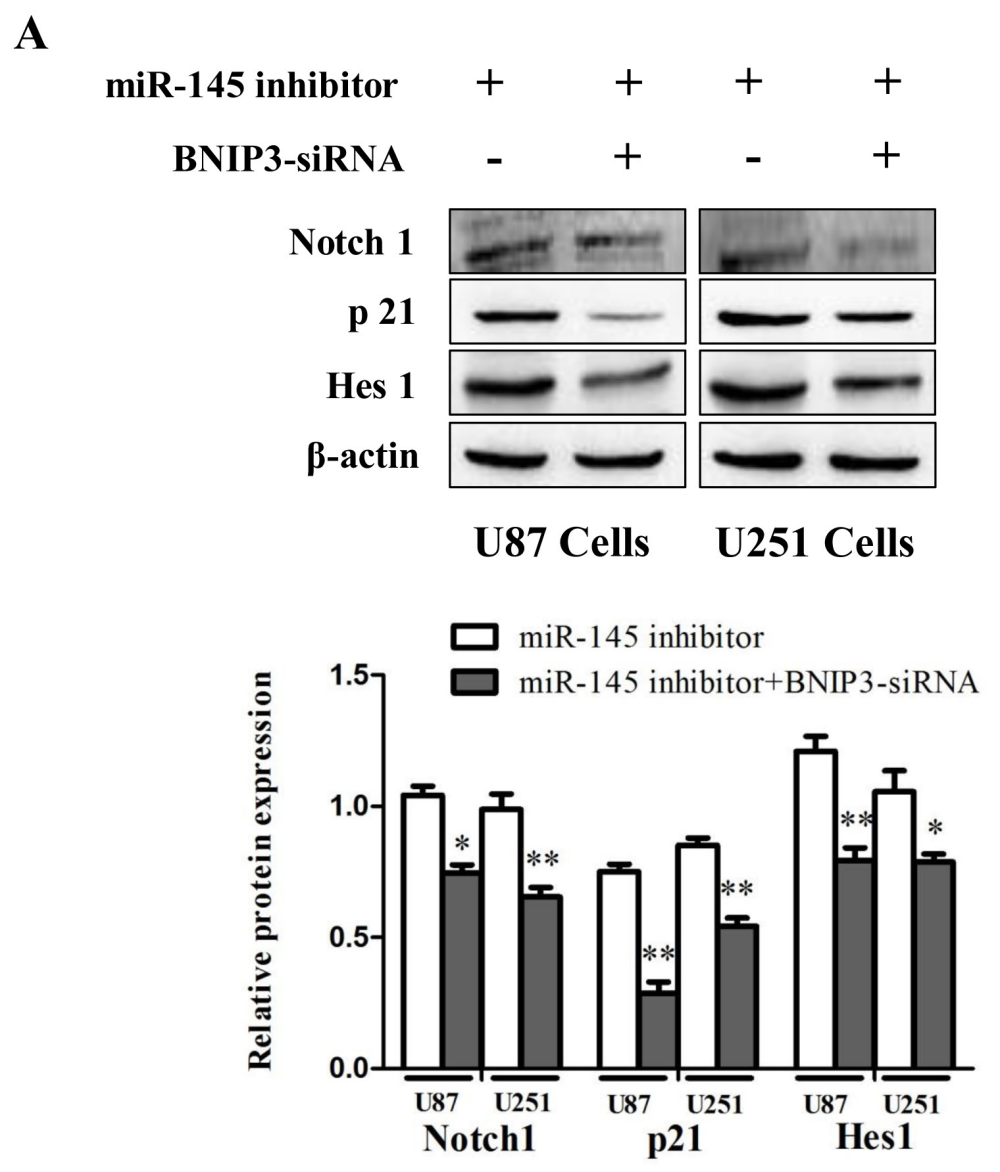
miR-145 can regulate Notch signaling pathway by targeting BNIP3 **(A)** Protein expression of Notch1, p21, Hes1 was determined by western blot analysis in U87 and U251 cells co-transfected with miR-145 inhibitor and BNIP3-siRNA, or with miR-145 inhibitor. *p < 0.05, **p < 0.01 versus control group.

To investigate the role of Notch signaling in glioma *in vivo*, protein levels of Notch1, p21 and Hes1 were analyzed by immunoblotting in human glioma samples (Figure 10A) and rat glioma tissues (Figure 10B). Since the Notch signaling was activated, we investigated whether miR-145 regulates the Notch pathway through BNIP3. Over-expression of miR-145 in U87 and U251 cells decreased protein levels of Notch1 and its downstream targets, p21 and Hes1 (Figure 11A). Correspondingly, Notch1, p21, and Hes1 protein levels increased in the presence of miR-145 inhibitor (Figure 11B). To explore whether the effect of miR-145 on the Notch pathway was related to BNIP3, protein levels of Notch1, p21, and Hes1 were analyzed in cells transfected with BNIP3 siRNA or the BNIP3 expression vector. As expected, the BNIP3 siRNA group exhibited increased protein levels (Figure 11C), while the BNIP3 expression vector group exhibited decreased protein levels (Figure 11D). To support these results, the protein expression of Notch1, p21, and Hes1 was also analyzed in U87 and U251 cells that were co-transfected with miR-145 inhibitor and BNIP3-siRNA, compared with miR-145 inhibitor. The results showed that there was a decrease in protein expression of Notch1 and its downstream target genes, p21 and Hes1 (Figure 12A). Taken together, these data indicate that miR-145 inhibits Notch signaling partly through BNIP3.

## DISCUSSION

Aberrant expression of miRNAs is closely related to tumorigenesis and contributes to the pathophysiology of glioblastoma [27, 28]. Several miRNAs regulate the activation of glioma cells, including miR-145. For example, miR-145 has been found to inhibit migration and invasion of gliomas stem cells by targeting ABCG2 [29]. In addition, miR-145 promotes the phenotype of Human Glioblastoma Cells selected for invasion [30]. However, the function of miR-145 in gliomas has not yet been elucidated. In this study, we demonstrate that the miR-145 expression is decreased in gliomas. Importantly, our results show that miR-145 induces apoptosis in glioma cells, suggesting that it functions as a tumor suppressor miRNA.

Target screening assays have linked miR-145 and BNIP3 [31-34]. Here, we present a strong evidence that miR-145 inhibits the expression of BNIP3 by binding to its 3′-UTR in glioma cells, and we demonstrate an inverse correlation between miR-145 and BNIP3 expression in glioma tissues. BNIP3 fails to associate with mitochondria in gliomas and promote cell death, due to its nuclear localization. These observations imply that BNIP3 acts as an oncogene and is involved in the regulation of miR-145-mediated apoptosis in gliomas.

Recent studies have focused on the effect of Notch signaling in glioma cells, which may have plasticity and respond to signals from their microenvironment [35-37]. Notch signaling is regulated by various miRNAs [38]. Indeed, miR-145 has been reported to play a pivotal role in Notch signaling [15]. In this study, we have identified BNIP3 as a target gene of miR-145. In addition, our results indicate that BNIP3 inhibits apoptosis of glioma cells by regulating the Notch signaling pathway. This conclusion is based on the following evidence. First, up-regulation of miR-145 and knockdown of BNIP3 decreased the protein expression of Notch1, Hes1, and p21 in glioma cells. Second, down-regulation of miR-145 and up-regulation of BNIP3 increased the protein expression of Notch1, Hes1, and p21 in glioma cells. Lastly, co-transfection of down-regulated miR-145 and knockdown of BNIP3 decreased the protein levels of the Notch1-regulated proteins.

Together, our results indicate that miR-145 increases apoptosis of glioma cells by directly inhibiting BNIP3, resulting in the inhibition of Notch signaling, and suggest that miR-145 may serve as a novel therapeutic target in malignant gliomas.

## MATERIALS AND METHODS

### Animal studies

Brain tumors were induced in male rats of the Sprague–Dawley strain, weighing 250–300 g, by stereotaxic injection of C6 cells in the left hemisphere. Rats were anesthetized with 10% chloral hydrate given intraperitoneally and immobilized on a stereotaxic unit. After disinfection and incision of the skin, a small burr hole (1 mm in diameter) was drilled at a position 3.5 mm left from the bregma, and C6 cells re-suspended in PBS (106 cells/2 μl) were implanted into the left hemisphere at a depth of 5.5 mm at an infusion rate of 1 μl/10 min. The needle was left in place 5 min after cell infusion, and then was slowly withdrawn. All tumor cell implantations were performed using a Hamilton syringe with a 26-gauge needle attached to the stereotaxic system.

After the tumor cell implantation, the tumor growth was monitored by brain magnetic resonance imaging (MRI) at 1, 2, and 3 weeks. Almost all rats survived until the end of the experiment, and were sacrificed 21–22 days post tumor implantation.

The study was carried out in accordance with the guidelines and regulations of the Chinese National Ministry of Science and Technology as well as National Ministry of Health. The animal experimental protocol (number: LLSC20150335) was approved by the University Animal Care and Use Committee.

### Brain magnetic resonance imaging (MRI)

MRI was performed on a clinical 1.5 Tesla Scanner (Signa Horizon, GE Healthcare, USA). The anesthetized animals were placed into an 8-channel wrist joint coil with an inner diameter of 4 cm for optimal covering of the brain. Conventional fast spin-echo pulse sequence (FSE) was used for T1 Weighed Imaging (T1WI; slice thickness 4 mm, field of view (FOV) 8×8cm, matrix 256×192, repetition times/echo time (TR/TE) 300/9.2 ms). For T2WI, the conditions were the following: slice thickness 4 mm, FOV 8×8cm, matrix 256×192, TR/TE 2000/22 ms. Scans were completed 5 min after injection of GdDTPA at a concentration of 0.05 mmol Gd.

### Histopathology

Tissues were fixed with 4% paraformaldehyde (24 h), and embedded in paraffin blocks for routine histology. Hematoxylin and eosin (H&E) and immunohistochemistry (IHC) staining was performed according to a standard procedure. Mouse monoclonal BNIP3 antibody (Bioss, China) for IHC was used at a 1:300 dilution. The pathological changes were assessed and photographed under an Olympus BX-51 microscope.

### Frozen tumor sections

The left brain was fixed in 4% formaldehyde (24 h), and dehydrated in a 20% and 30% sucrose solution. The brain was embedded, and 1 mm sections were prepared.

### Patient tissue samples and cell lines

Patient tissue samples (n =29) were collected from the neurosurgery department of Anhui provincial hospital and the First Affiliated Hospital of Anhui Medical University (HeFei, China). A total of 19 glioma samples were used for this study (WHO I/II, n=11 and WHO III/IV, n=8) and the10 normal brain tissues derived from the 10 patients who underwent a partial excision of the brain tissue due to traumatic brain injury or intracerebral hemorrhage. The study was approved by the Research Ethics Committee of the Anhui Medical University, and informed consent was obtained from all patients.

Human U251 and U87 glioblastoma cells, and rat C6 glioma cells were from the First Affiliated Hospital of Anhui Medical University, and were grown in DMEM high glucose medium (Hyclone) with 8% FBS at 37°C in a humidified atmosphere containing 5% CO_2_. Stable cell lines were derived from U251 and U87 cells by transfection with miR-145 mimics, inhibitor, BNIP3 siRNA, BNIP3-pEX-2 expression vector, or corresponding control RNA using Lipofectamine 2000 for 48 h.

### Quantitative real-time PCR

Total RNA was extracted from brain tissues or glioma cells using TRIzol reagent (Invitrogen) and reverse transcribed to cDNA using a Thermoscript RT-PCR reagent kit. Gene expression was determined using cDNA SYBR-Green real-time PCR Master Mix by quantitative real-time PCR (Takara). The mRNA ratio of the target gene to GAPDH was calculated by using the 2−ΔΔCt formula. The experiments were performed at least three times using three different templates. The primers used were: β-actin (forward: 5′-CCCATCTATGAGGGTTACGC-3′; reverse: 5′-TTTAATG TCACGCACGATTTC-3′), GAPDH (forward: 5′-ACCACAGTCCATGCCATCAC-3′ reverse: 5′-TCCACCACCCTGTTGCTGTA-3′), BNIP3 (forward: 5′-TCCAGCCTCGGTTTCTATTT-3′ reverse: 5′-AGCTCTTGGAGCTACTCCGT-3′).

### Western blotting

Brain tissues and cultured cells were lysed with RIPA lysis buffer and protein concentration was measured using a BCA protein assay kit (Boster, China). The extracts (20 or 40 μg) were then separated on 8% or 12% SDS-PAGE gels and transferred to PVDF membranes (Millipore, Billerica, MA, USA). The membranes were blocked in 5% nonfat milk in TBST (3 h), and incubated overnight with primary antibodies, followed by corresponding horseradish peroxidase-conjugated anti-rabbit secondary antibodies (1 h). The protein bands were visualized using an ECL-chemiluminescent kit (ECL-plus, Thermo Scientific). Quantitative densitometric analyses of immunoblotting images were performed using Image J software. The experiment was repeated for three times. Rabbit monoclonal antibodies against Bax, Bcl-2, Caspase-3, and p21 (Abcam, USA) were used at 1:600 dilution, rabbit monoclonal antibody against Notch 1 Hes1 (Cell Signaling Technology, USA) was used at 1:600, anti-BNIP3 antibodies (Boster, China), anti-β-actin and anti-PCNA (ZSGB-BIO, China) were diluted 1:300

### Hoechst staining

After infection, cells were fixed with 4% paraformaldehyde for 15 min, washed with PBS, and stained with Hoechst 33342 at 37°C for 20 min in the dark. Cells were rinsed with PBS and mounted on coverslips, which were photographed using Olympus BX-51 microscope.

### Flow cytometry

Cells were harvested by centrifugation at 1800 rpm for 5 min after 48 h of infection, and washed twice with PBS. The washed cells were re-suspended in 400 μl of Annexin. The cells were stained with 5 μl of Annexin V-FITC at 4 °C for 15 min in dark and then with 10 μl of PI at 4 °C in dark for 5 min. The analyses were performed on flow cytometer (Beckman Coulter); each experiment was conducted in triplicate.

### Immunofluorescence staining

Transfected cells or frozen brain sections were fixed in acetone for 15 min, washed with PBS, and blocked with 10% BSA for 10 min at room temperature. Cells were incubated for 12 h with a polyclonal antibody to human BNIP3 (1:100 dilution, Bioss, China) containing 1% BSA at dark, and then with secondary antibody (1 h). The cells were mounted with DAPI (Beyotime). Immunofluorescence was examined using an Olympus BX-51 microscope; BNIP3 was shown as green fluorescence.

### Tunel staining

Apoptosis was evaluated by Tunel staining according to the kit manufacturer’s protocol (KeyGEN BioTECH, Najing, China). Briefly, glioma cells were fixed with 4% formaldehyde at room temperature for 30 minutes, permeabilized with 1% Triton X-100 solution for 5 minutes, and incubated with 50 μl of TdT enzyme for 60 minutes at 37°C. Cells were washed with PBS, incubated with 50 μl of Streptavidin-Fluorescein for 30 min at 37°C, mounted with DAPI, and examined using Olympus BX-51 microscope

### Plasmid transfection and luciferase assays

PYr-MirTarget-BNIP3 3′-UTR plasmid was obtained from Yin-grun Biotechnology (Changsha, Hunan, China). U87 and U251 cells were plated in 96 well plates, and then transfected with 200 ng plasmid and miR-145 mimics at 50 nM concentration for 24 h. Dual luciferase assay was performed to measure the firefly luciferase conjugated to the 3′-UTR normalized to Renilla luciferase activity (Promega).

### Statistical analysis

The results were analyzed using the SPSS software (version 17.0). All experiments were performed independently at least three times. Results are expressed as means ± SD. Statistical analysis was performed using an analysis of variance (ANOVA) and Student’s t-test. Statistical values of P<0.05 were considered to be statistically significant.

## Abbreviations

GBM: glioblastoma multiforme
miRNAs: microRNAs
Hes: hairy/enhancer of split
Hey: Hes-related with YRPW motif
BNIP3: bcl2/adenovirus E1b interacting protein 3
HDAC1: histone deacetylase 1
PSF: PTB-associating splicing factor
